# Comparison of paediatric infectious disease deaths in public sector health facilities using different data sources in the Western Cape, South Africa (2007–2021)

**DOI:** 10.1186/s12879-023-08012-6

**Published:** 2023-02-22

**Authors:** K. Kehoe, E. Morden, T. Jacobs, N. Zinyakatira, M. Smith, A. Heekes, J. Murray, D. M. le Roux, T. Wessels, M. Richards, B. Eley, H. E. Jones, M. T. Redaniel, M. A. Davies

**Affiliations:** 1 Centre for Infectious Disease Epidemiology and Research, School of Public Health and Family Medicine, University of Cape Town, Cape Town, South Africa; 2 Population Health Sciences, Bristol Medical School, University of Bristol, Bristol, UK; 3 Health Intelligence Directorate, Western Cape Government Health, Cape Town, South Africa; 4 Division of Public Health Medicine, School of Public Health and Family Medicine, University of Cape Town, Cape Town, South Africa; 5 Department of Paediatrics and Neonatology, Paarl Hospital, Cape Town, South Africa; 6 Department of Paediatrics and Child Health, Red Cross War Memorial Children’s Hospital, University of Cape Town, Cape Town, South Africa; 7 District Paediatrician Cape Town Metro East, Department of Paediatrics and Child Health, Tygerberg Hospital, Stellenbosch University, Stellenbosch, South Africa; 8 Faculty of Health Sciences, University of Cape Town, Cape Town, South Africa; 9 Paediatric Infectious Diseases Unit, Red Cross War Memorial Children’s Hospital, Cape Town, South Africa; 10 The National Institute for Health Research Applied Research Collaboration West (NIHR ARC West) at University Hospitals Bristol and Weston NHS Foundation Trust, Bristol, UK

**Keywords:** Paediatric infectious disease deaths, Data comparison, Data completeness, South Africa

## Abstract

**Background:**

Routinely collected population-wide health data are often used to understand mortality trends including child mortality, as these data are often available more readily or quickly and for lower geographic levels than population-wide mortality data. However, understanding the completeness and accuracy of routine health data sources is essential for their appropriate interpretation and use. This study aims to assess the accuracy of diagnostic coding for public sector in-facility childhood (age < 5 years) infectious disease deaths (lower respiratory tract infections [LRTI], diarrhoea, meningitis, and tuberculous meningitis [TBM]) in routine hospital information systems (RHIS) through comparison with causes of death identified in a child death audit system (Child Healthcare Problem Identification Programme [Child PIP]) and the vital registration system (Death Notification [DN] Surveillance) in the Western Cape, South Africa and to calculate admission mortality rates (number of deaths in admitted patients per 1000 live births) using the best available data from all sources.

**Methods:**

The three data sources: RHIS, Child PIP, and DN Surveillance are integrated and linked by the Western Cape Provincial Health Data Centre using a unique patient identifier. We calculated the deduplicated total number of infectious disease deaths and estimated admission mortality rates using all three data sources. We determined the completeness of Child PIP and DN Surveillance in identifying deaths recorded in RHIS and the level of agreement for causes of death between data sources.

**Results:**

Completeness of recorded in-facility infectious disease deaths in Child PIP (23/05/2007–08/02/2021) and DN Surveillance (2010–2013) was 70% and 69% respectively. The greatest agreement in infectious causes of death were for diarrhoea and LRTI: 92% and 84% respectively between RHIS and Child PIP, and 98% and 83% respectively between RHIS and DN Surveillance. In-facility infectious disease admission mortality rates decreased significantly for the province: 1.60 (95% CI: 1.37–1.85) to 0.73 (95% CI: 0.56–0.93) deaths per 1000 live births from 2007 to 2020.

**Conclusion:**

RHIS had accurate causes of death amongst children dying from infectious diseases, particularly for diarrhoea and LRTI, with declining in-facility admission mortality rates over time. We recommend integrating data sources to ensure the most accurate assessment of child deaths.

**Supplementary Information:**

The online version contains supplementary material available at 10.1186/s12879-023-08012-6.

## Introduction

Monitoring childhood mortality is important to assess factors related to child survival such as access to and quality of healthcare, safety, nutrition and protection [1, 2]. This closely aligns with the third Sustainable Development Goal that by 2030 all countries should reduce under-five mortality rate to < 25 deaths/1000 live births [3]. Accurate monitoring of the contribution to mortality of infectious diseases, including lower respiratory infections (LRTI), diarrhoea, meningitis, and tuberculous meningitis (TBM), is thus vital to monitor progress, as these preventable deaths are responsible for a substantial proportion of child mortality in resource-limited settings [4–7]. Data for these infectious diseases are often outdated or scarce, especially at lower geographic levels within these settings, warranting the need to better understand the current burden.

Childhood mortality monitoring through death notification systems may have several disadvantages including the limited use of these systems in many settings and that there may be up to 2 year delays in releasing these data, making them unhelpful for real-time use [5, 8]. In addition, not all child deaths may be registered [9], resulting in low completeness [4]. Routinely collected health datasets may provide more rapid child mortality estimates and the limitations of the several different routine health data sources available can be reduced by integrating and comparing them [10].

We compared the completeness of death data for children recorded as dying from infectious diseases in the Western Cape, South Africa, using three death data sources as follows: (i) routine health information systems (RHIS) data which captures key information for each admission, including the unique patient identifier, patient demographics and hospital administration data, and discharge codes and summaries [11]; (ii) Child Healthcare Problem Identification Programme (Child PIP) which is a child death audit review process of all in-hospital child (0–18 years) deaths in public health facilities that participate voluntarily [12, 13]; and (iii) Death Notification (DN) Surveillance which includes all deaths, in- or out-of-hospital, recorded on DN forms [14]. These three data sources are integrated and linked in the Western Cape Provincial Health Data Centre (WCPHDC) through a unique patient identifier that is used across public health services in the province [11]. RHIS includes every electronically recorded in-facility child death but does not typically include cause of death, but rather diagnostic coding of the reason for admission during which the death occurred. In contrast, there may be under-reporting or missing data on all in-facility child deaths in DN Surveillance and Child PIP, but these capture recorded causes of death from notification forms and audits respectively, making their cause of death data more accurate. Child death audit review systems, used globally [15], define causes of death similarly to DN, but also identify modifiable factors contributing to these deaths [12, 13].

In addition to comparing completeness of the different death data sources, we assessed the accuracy of RHIS recorded diagnostic codes vs. those in DN Surveillance and Child PIP. We determined the admission mortality rates for in-facility infectious disease deaths in the public sector across the Western Cape province and by district/sub-district.

## Methods

### Setting study population

The Western Cape province consists of six districts: Cape Town Metro, Cape Winelands, Central Karoo, Garden Route, Overberg, and West Coast. The province had an estimated seven million people in 2020 [16] of whom 8% (563,590) were children aged 0–4 years [17]. About 80% of the child population utilise public-sector services. The Western Cape Department of Health manages 52 hospitals and 272 primary care clinics, and the City of Cape Town manages an additional 82 clinics [11]. We included all children hospitalised at age < 5 years, including neonates, at a public health facility within the Western Cape province from 2007 to 2021.

### Data sources

We use the following data sources that are consolidated within the WCPHDC (Table 1): RHIS, Child PIP, and DN Surveillance data. The following infectious disease death categories were identified and used in the analysis: LRTI, diarrhoea, meningitis, and TBM (Additional file 1: Table S1). Unless otherwise stated, all totals reported in the results are related to these four infectious diseases only. We also identified an ‘other infectious disease’ category when looking at cause of death coding (Additional file 1: Table S1). This was utilised in the concordance analysis to determine if the diagnostic coding in the RHIS would detect an infectious disease e.g. HIV, even if an International Classification of Diseases 10th Revision (ICD-10) for the four infectious diseases was absent. The list of ICD-10 codes for inferring each infectious disease were determined in consultation with clinicians including paediatric infectious disease specialists.

**Table 1:** Overview of data sources used for comparing infectious disease deaths in children under five years

	Sources utilised from the Western Cape Provincial Health Data Centre A single consolidated environment which houses individuated patient-level health data for the Western Cape		
	Routine hospital information systems	Child Healthcare Problem Identification Programme	Death Notification (DN) Surveillance
Brief description	Routine hospital information systems in the province capturing key information for each admission, including the unique patient identifier, patient demographics and hospital administration data, and discharge codes and summaries	An audit process of all in-hospital child (0–18 years) deaths in public health facilities that participate voluntarily	All deaths, in- or out-of-hospital, are recorded once the DN forms have been captured by the DHA. These data were leveraged for DN with cause of death data being shared with the DoH
Time period	01/01/2007–01/12/2021	23/05/2007–08/02/2021	01/01/2010–01/12/2013
Coverage	All public hospitals capturing in-facility deaths since the date when electronic admission and discharge data were networked into the WCPHDC.*	Selected public health facilities that voluntarily participate, thereby only capturing in-facility deaths in participating facilities	All notified deaths in the province, in- or out-of-hospital. For this study, we restricted to in-facility deaths
Source of death reporting	Admission ICD-10 coding (primary, subsidiary and secondary 1–10) from clinical records	Cause of death as determined by clinical audit	Cause of death as recorded on DN form
Variables	Date of death, ICD-10 codes (to determine proxy cause of death), and hospital admission date	Date of death, cause of death, and hospital admission date	Date of death and cause of death
Frequency of data source updates to the WCPHDC	Daily	Approximately annually	No longer provided
Advantages	Complete and timeous recording of all deaths in facilities with electronic data capture as every separation requires an entry	Reports in depth information on child deaths, including modifiable factors. Reports are available timeously	Includes all deaths (public and private health sector, and out-of-hospital deaths) associated causes
Disadvantages	Only available for public health facilities and in-hospital deaths. The cause of death is not explicitly coded, but has to be inferred from the diagnostic code for the admission during which the death occurred	Not all public health facilities participate (voluntary). Private sector death information is not included	Cannot be used for short/medium term planning due to the reporting delay (2 years) Contributing or modifiable factors are not recorded Deaths are underreported (not reported or form does not reach the DHA), incomplete or incorrect. Deaths are also not available for foreign nationals without a South African identification number. Data are collated nationally and individual cause of death data is not provided to provincial health departments. Aggregate mortality data are only provided at the level of the province and not for smaller geographic areas

*DHA* Department of Home Affairs, *DoH* Department of Health, *DN* Death Notification, *ICD-10* International Classification of Diseases 10th Revision

*In-facility death reporting was achieved incrementally since 2007 as hospitals became networked in the province

#### Western Cape Provincial Health Data Centre (WCPHDC)

The WCPHDC is a single consolidated environment that links individuated patient-level health data from different health information systems using a unique identifier available in all patient administration systems in public health facilities in the Western Cape, South Africa [11]. Data sources include hospitals (both inpatient and outpatient visits), primary healthcare facilities (outpatient visits), diagnostic laboratories, pharmacies, disease management systems, community data, partner systems, and mobile health systems [11].

The primary focus of the WCPHDC is to provide data for service delivery and support clinical care. Using these data, encounters (health service contacts e.g., outpatient visits or admissions) and health conditions e.g., HIV, tuberculosis, pregnancy and cascades (health care utilisation and outcomes of health conditions) are identified. Health conditions are inferred with different levels of certainty using multiple data sources (e.g. diagnostic or disease specific laboratory tests, medication, disease management system data and diagnostic [ICD10] codes). Most of these data are updated and linked daily with other sources, which are updated weekly, monthly, quarterly, or periodically depending on the data source [11]. Integration of this data improves the data quality, reducing the concerns regarding a single data source.

#### Routine hospital information systems (RHIS)

RHIS (known as Clinicom [11] and Electronic Continuity of Care Record [ECCR]) are used in hospitals in the province to record patient demographics, admissions, discharge summaries, diagnosis codes and hospital administration data against each unique identifier [11]. Reporting was achieved incrementally since 2007 as hospitals became networked in the province [18]. Each hospitalisation and the corresponding outcome (death, transfer to another facility or home discharge) is recorded. We identified an infectious disease by the presence of a relevant ICD-10 code across any of the recorded fields (i.e. primary, subsidiary, or first ten recorded secondary codes). For in-facility deaths, the cause of death is not specifically captured, and so we used ICD-10 diagnosis codes identified during the admission as the proxy cause of death (Table 1). In a child who is transferred between wards or facilities, there may be several records related to the admission when death occurred with multiple ICD-10 codes captured across these records. Using the three infectious disease ICD-10 codes closest to the event, a single proxy cause of death was identified with primary causes in turn outranking subsidiary or secondary causes and then codes recorded closest to the event outranking those recorded earlier.

#### Child Healthcare Problem Identification Programme (Child PIP)

Child PIP was established in 2005 to audit all in-facility deaths in children (0–18 years) in South Africa and improve healthcare provision [12, 13] (Table 1). The mortality review process involves detailed steps in which (i) the child death is reviewed within 24 h, (ii) a nurse and doctor present cases at weekly or monthly mortality meetings, and, (iii) data management and analysis identifies trends and makes recommendations [13]. The cause of death is identified through a clinical audit process. As Child PIP is voluntary, uptake varies within the Western Cape, with 37 out of 43 hospitals (86%) currently participating. The individual patient-level data are collected at facility level to review practices, and reported into a national Child PIP database to inform policies nationally. Key social and nutritional data are collected to describe the circumstances surrounding the death. Critical to the process is identifying potential modifiable factors, divided into two categories: person (e.g., administrator, clinical personnel, caregiver) and place (e.g., ward, home, referring facility).

#### Death notification (DN) surveillance

The Western Cape Department of Health (DoH), City of Cape Town and South African Medical Research Council Burden of Disease Research Unit together developed a Death Notification (DN) Surveillance system to monitor district and subdistrict mortality and causes of death [14] (Table 1), leveraging vital registration data reported to the South African Department of Home Affairs (DHA). Six district information offices collected copies of DN forms from the local DHA offices [14]. Sociodemographic and cause of death information from the DN was captured with data cleaning and mortality reports for the province produced. Patient-level cause of death data was shared with the Western Cape DoH between 2010 and 2013. This system was discontinued from 2014.

### Data comparison and analysis

Data were linked across the RHIS, Child PIP and DN Surveillance data using the unique identifier, for the four infectious diseases of interest for the respective time periods that data were available. If dates of death differed by more than 30 days between datasets or did not align to an admission date in RHIS, the child was dropped from the dataset (three children). Where cause of death differed across datasets, we applied the following hierarchy in attributing an overall cause of death to determine mortality estimates: (1) Child PIP (most reliable) as causes of death are identified through an audit process; (2) DN ranked second as cause of death is identified by the attending clinician at the time of death, but without an audit process; and (3) RHIS ranked third as cause of death had to be inferred from admission diagnosis codes.

We compared data to determine the number of de-duplicated infectious diseases deaths across all sources. We determined the percentage of each of the infectious disease deaths present in Child PIP and DN Surveillance vs. RHIS. To understand the accuracy of RHIS causes of death, we determined the level of agreement between RHIS and each of Child PIP and DN Surveillance. To test the level of agreement, we calculated the Kappa statistic for the four infectious diseases of interest, with the agreement for values of 0.81–1.00 being considered “almost perfect”, 0.61–0.80 “substantial”, 0.41–0.60 “moderate”, 0.21–0.40 “fair” and 0.01–0.20 “none to slight” [19].

We calculated admission mortality rates for the province and each district/subdistrict (based on child’s place of residence) over time using the de-duplicated, integrated dataset that included all sources. Admission mortality rate is defined as infectious disease deaths occurring in a health facility divided by live birth population estimates, presented per 1000 live births with 95% confidence intervals. The population denominator was estimated from population estimates as 80% of live births [20], i.e. the estimated proportion of the population using public sector services in the province or respective district/sub-district. Since the number of live births per Cape Town Metro sub-district was not available, as births are mapped to Home Affairs Offices rather than place of residence, we estimated the sub-district live births denominator based on the proportion of infants among the total population for that sub-district. Live births from the population estimates were only available until 2020, so the comparison was limited to 2007–2020. For district and sub-district comparisons, 2019 death and population data was used, as this was the last pre-pandemic year and data thereafter are confounded by several pandemic related factors [21].

Data were cleaned and coded in SQL and analysed using Excel and R Studio.

### Ethics

This study was approved by the Human Research Ethics Committee in the Faculty of Health Sciences at the University of Cape Town (HREC REF 197/2021).

## Results

Using all three data sources, there were 217,899 admissions for one of the four infectious disease admissions among children under 5 years from 2007 to 2021 with a total of 1947 deaths recorded among these children (~ 1% of hospital admissions among children under five years with an infectious disease diagnosis) (Table 2). Around 1661 children died (85% of all deaths) from one of the four infectious diseases included in this study. Of the remaining 286 deaths, most had either missing cause of death and missing ICD-10 code or were attributed to sepsis or perinatal factors such as low birth weight and prematurity. The highest proportion of infectious disease deaths was in children aged 28 days to one year (1000 deaths, 60%). Most deaths occurred in the two large tertiary hospitals in the province which account for 39% of all infectious admissions (713 deaths [43% of infectious disease deaths] and 596 deaths [36% of infectious disease deaths] in these two hospital respectively). The highest proportion of infectious disease admissions at any other hospital accounted for ≤ 4%. Most primary diagnosis codes for infectious disease deaths in RHIS were coded by a registrar or consultant (1430 deaths, 86%), rather than a data clerk, whereas less than a third of subsidiary codes were coded by a registrar or consultant (532 deaths, 32%).

**Table 2:** Summary of infectious disease deaths among 217,899 children admitted for an infectious disease in public sector facilities in the Western Cape, 2007–2021

Variable	Total (%)
Total number of deaths	1947 (~ 1% of infectious disease admissions)
Infectious disease deaths (LRTI, diarrhoea, TBM or meningitis)	1661 (85% of total deaths)
Other causes of death (missing cause of death or ICD-10 code, sepsis, or perinatal factors [low birth weight or prematurity])	286 (15% of total deaths)
Age among infectious disease deaths	
< 28 days	202 (12%)
28 days to 1 year	1000 (60%)
> 1 to < 5 years	459 (28%)
Location	
Hospital A	713 (43% of infectious disease deaths)
Hospital B	596 (36% of infectious disease deaths)
Other hospitals	352 (21% of infectious disease deaths)
ICD-10 coding	
Primary diagnosis coding by registrar or consultant	1430 (86% of infectious disease deaths)
Subsidiary diagnosis coding by registrar or consultant	532 (32% of infectious disease deaths)

### Data source comparison

**Fig. 1:**
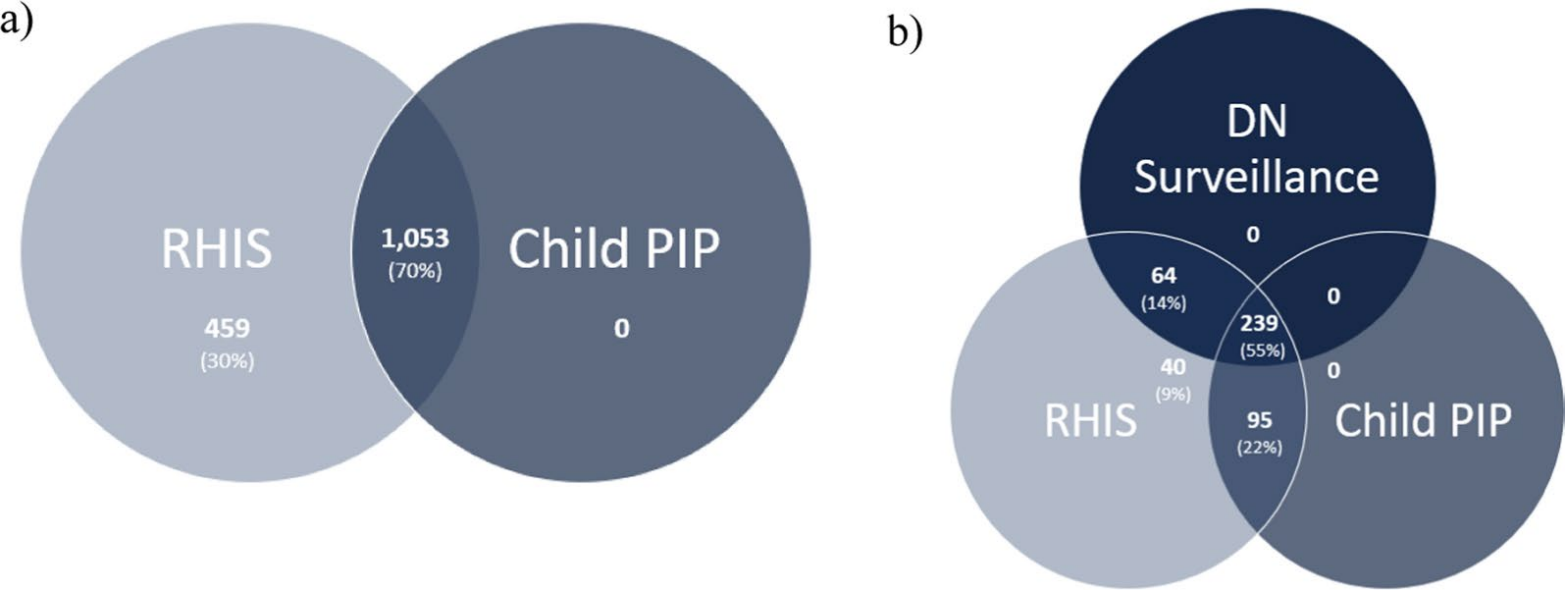
Venn diagrams triangulating infectious disease deaths across data sources for the time periods that the data sources were available: **a** RHIS and Child PIP from 23/05/2007–08/02/2021 (1512 deaths) and **b** RHIS, DN Surveillance and Child PIP from 2010 to 2013 (438 deaths)

During the time period for which RHIS and Child PIP data were both available (23/05/2007–08/02/2021), 1512 infectious deaths were recorded across these two data sources (Fig. 1a). All of these were recorded in RHIS, and Child PIP was 70% complete (1053 deaths).

When comparing infectious disease deaths across all available data sources for the period when all three sources where available (438 deaths from 2010 to 2013), DN Surveillance was 69% complete (303 deaths identified, of which 239 were also recorded in Child PIP while 64 were recorded in DN Surveillance and RHIS only) (Fig. 1b). An additional 22% of deaths were identified in RHIS and Child PIP but not DN Surveillance (95 deaths), while the remaining 9% were in RHIS alone (40 deaths).

### Cause of death agreement

Of the 1186 deaths recorded in Child PIP (considered most reliable for cause of death) from May 2007–February 2021, 564 (48%) were due to one of the four infectious diseases of interest. Although all of these deaths were recorded in RHIS, 3% (16 deaths) could not be identified as an infectious disease death based on RHIS alone. An additional 2% (10 deaths) were identified as an infectious disease death but not as being due to one of the four infectious diseases of study (Table 3). The concordance between Child PIP and RHIS: diarrhoea (92%), LRTI (84%), meningitis (77%), and TBM (74%). The overall level of agreement for the four infectious diseases was almost perfect (Kappa statistic 0.810; 95% CI 0.766–0.855). For Child PIP causes of death, a greater proportion of mismatches were detected for meningitis and TBM, whereas a greater number of mismatches was detected for LRTI as this is more common; all due to the absence of the corresponding diagnostic code in RHIS.

**Table 3:** Comparison of Routine Health Information Systems and Child Healthcare Problem Identification Programme causes of death among children admitted for diarrhoea, lower respiratory tract infections, meningitis or tuberculous meningitis in the Western Cape (May 2007–February 2021)

Child PIP cause of death	RHIS cause of death						
	Diarrhoea	LRTI	Meningitis	TBM	Other infectious disease	Other condition	Total
Diarrhoea	**119 (92%)**	6 (5%)	1 (1%)	0	0	3 (2%)	129 (100%)
LRTI	29 (9%)	**285 (84%)**	5 (1%)	0	9 (3%)	10 (3%)	338 (100%)
Meningitis	1 (2%)	7 (11%)	**48 (77%)**	3 (5%)	1 (2%)	2 (3%)	62 (100%)
TBM	2 (6%)	2 (6%)	4 (11%)	**26 (74%)**	0	1 (3%)	35 (100%)
Total	151	300	58	29	10	16	564

Table includes all deaths that were recorded as being due to diarrhoea, LRTI, meningitis or TBM in Child PIP. Row percentages are shown. Bold values are to highlight the level of agreement of each infectious disease, respectively

*Child PIP* Child Healthcare Problem Identification Programme, *LRTI* lower respiratory tract infections, *RHIS* Routine Health Information Systems, *TBM* tuberculous meningitis

Of the 542 deaths in DN Surveillance from 2010 to 2013, 132 (15%) were due to one of the four infectious diseases of interest. Of these, 128 (96%) were identified as being due to an infectious disease of interest in RHIS (Table 4). The overall level of agreement for the four infectious diseases was moderate (Kappa statistic 0.782; 95% CI 0.690–0.875). The best agreement between DN Surveillance and RHIS was for diarrhoea (98%) and LRTI (83%). TBM (45%) and meningitis (56%) agreement was lower mostly due to the absence of the corresponding diagnostic code in RHIS. Again, the greater number of mismatches was detected for LRTI cause of death in DN Surveillance.

**Table 4:** Comparison of Routine Health Information Systems and Death Notification Surveillance causes of death among children admitted for diarrhoea, lower respiratory tract infections, meningitis or tuberculous meningitis in the Western Cape (2010–2013)

DN Surveillance cause of death	RHIS cause of death					
	Diarrhoea	LRTI	Meningitis	TBM	Other condition	Total
Diarrhoea	**52 (98%)**	1 (2%)	0	0	0	53
LRTI	8 (14%)	**49 (83%)**	0	0	2 (3%)	59
Meningitis	0	3 (27%)	**5 (45%)**	2 (18%)	1 (9%)	11
TBM	1 (11%)	0	2 (22%)	**5 (56%)**	1 (11%)	9
Total	61	53	7	7	4	132

Table includes all deaths that were recorded as being due to diarrhoea, LRTI, meningitis or TBM in DN Surveillance. Row percentages are shown. Bold values are to highlight the level of agreement of each infectious disease, respectively

*DN* Death Notification, *LRTI* lower respiratory tract infections, *RHIS* Routine Health Information Systems, *TBM* tuberculous meningitis

### Admission mortality rates

**Fig. 2:**
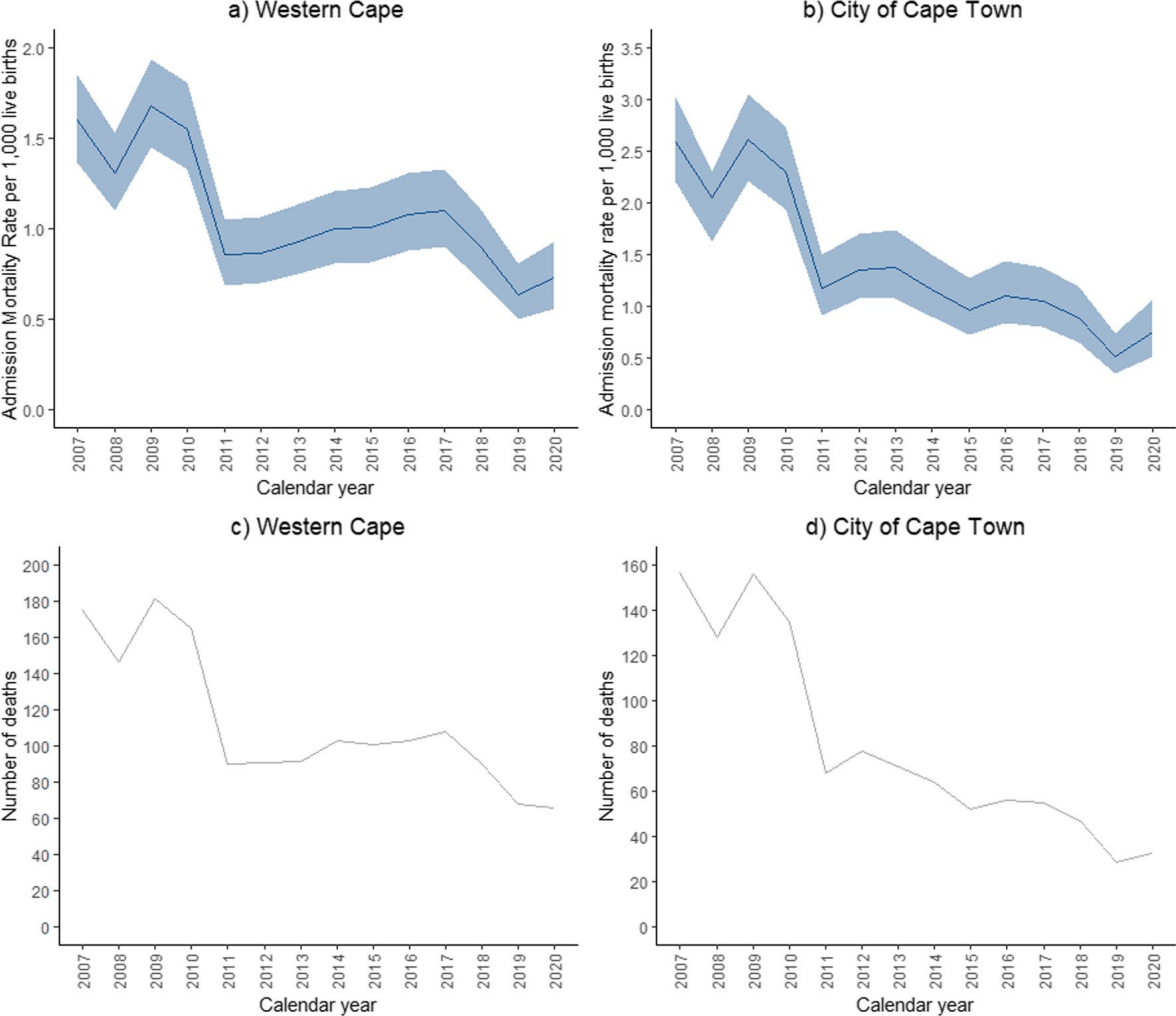
Admission mortality rates per 1000 live births mapped to child’s residence, with 95% confidence intervals represented by the shaded areas for **a** the Western Cape and **b** the City of Cape Town and the total number of infectious disease deaths for **c** the Western Cape and **d** the City of Cape Town for all four infectious disease deaths combined using the de-duplicated dataset. Population estimates were only available until 2020, so the comparison was limited to 2007–2020

Using the combined de-duplicated dataset, admission mortality rates for these infectious diseases in the Western Cape more than halved between 2007 and 2020 (1.60 [95% CI: 1.37–1.85)] deaths per 1000 live births [2007] and 0.73 [95% CI: 0.56–0.93] deaths per 1000 live births [2020]) (Fig. 2a). Of the 1661 infectious disease deaths from 2007–2021, 1168 (70%) were in the Cape Town Metro (Fig. 2b). Admission mortality rates for the four infectious diseases of interest in Cape Town Metro decreased by almost fourfold from 2.59 (95% CI: 2.20–3.03deaths per 1000 births [2007] to 0.76 (95% CI: 0.52–1.07) deaths per 1000 live births [2020]. Only 2% (25 deaths) of infectious disease deaths could not be mapped to a child’s place of residence in the province (either outside the province or missing).

**Fig. 3:**
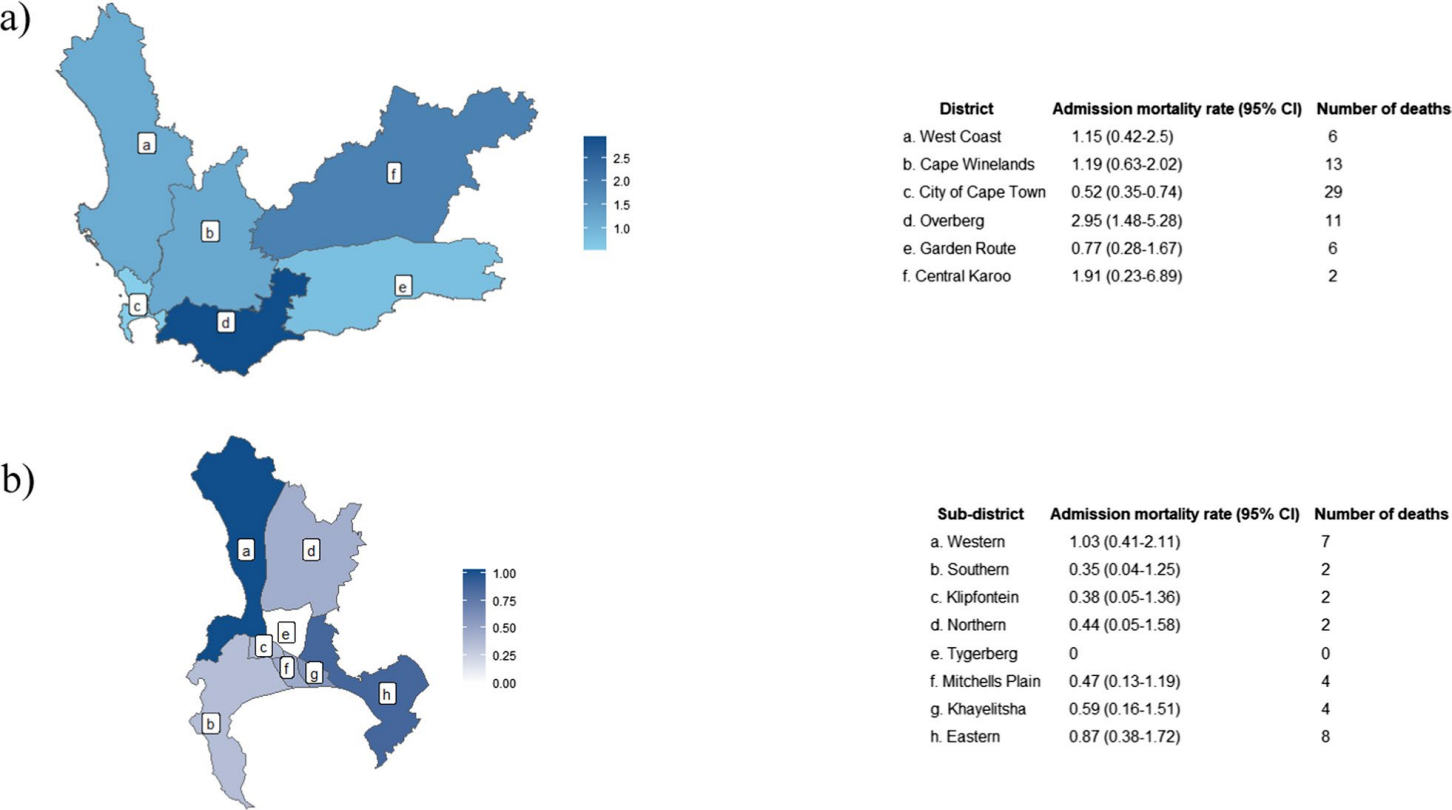
Admission mortality rates per 1000 live births (95% confidence interval) and number of deaths mapped to child’s residence using the combined de-duplicated dataset and population estimates as the denominator for the four infectious disease deaths of interest in 2019 by **a** all Western Cape districts and **b** the City of Cape Town sub-districts. 2019 death and population data were used, as this was the last pre-pandemic year and data thereafter are confounded by several pandemic related factors

In 2019, the admission mortality rates were highest in Overberg and Central Karoo (Fig. 3a). Admission mortality rates in the other four districts were around one or lower death per 1000 live births. For the City of Cape Town, where the highest number of deaths were recorded, the highest admission mortality rate was in the Western sub-district with other sub-districts below one death per 1000 live births (Fig. 3b).

## Discussion

We believe this is the first study to compare and synthesize routinely collected in-facility child death data in the public sector in South Africa. We were able to comprehensively evaluate the accuracy of in-facility diagnostic coding for children who die from infectious diseases and demonstrated that data linkage and consolidation across multiple sources improved the accuracy of in-facility infectious disease mortality estimates.

Completeness of Child PIP and DN Surveillance for the four infectious disease deaths recorded electronically in RHIS was approximately 70%. While causes of death in RHIS rely on inference from the diagnosis code for the relevant admission, the level of agreement for the infectious cause of death with more accurate sources was generally high, specifically between Child PIP and RHIS (≥ 74%) and for diarrhoea and LRTI in both Child PIP and DN Surveillance (> 80%). The higher concordance may be because diagnoses of diarrhoea or LRTI can be made confidently based on symptoms only, whereas laboratory tests would usually be needed to confirm diagnoses of meningitis and TBM, and pathogen testing seldomly happens in this setting. Lower level of agreement for meningitis and TBM may be driven by suboptimal diagnosis, infrequent pathogen testing and incorrect diagnosis coding. Despite these limitations, the level of agreement across data sources provide reassurance that RHIS has reasonable accuracy of diagnostic coding for children who die. It is thus a useful and timely source of child death data, since RHIS is updated daily, whereas audit data sources (Child PIP) are only provided and integrated into the WCPHDC periodically and DN data is very delayed, not available at district/sub-district level and currently not available to health services, preventing linkage to other individual patient data. While low completeness of electronic ICD-10 coding was reported in a large tertiary hospital in the province [22], we found more accurate and complete diagnostic coding in patients who die, which has also been shown in other settings [8]. Our findings suggest that data comparison improves validity over using a single data source, which is consistent with other public health studies utilising triangulation or integration of routine health data sources, individual- or population-level, focusing on various health conditions [23–26].

The admission mortality rates for the four infectious diseases decreased significantly over the 14-year period, somewhat plateauing from 2014/15 with a slight reduction noted from 2019. This downward trend has been observed across the province for the individual infectious diseases where data are available, including pneumonia [27] and diarrhoea [28]. These declines may be due to: (i) improved prevention of HIV from mother-to-child and increased antiretroviral therapy coverage [29], (ii) the introduction of pneumococcal conjugate and rotavirus vaccines in 2009 [30], resulting in better prognoses for sick children and decreased pressure on health services due to a reduction in admissions, thus resulting in better outcomes, and/or iii) the improvement of services by standardising clinical governance through systems like Child PIP and child death reviews [12, 13, 31]. When comparing across districts within the Western Cape, childhood mortality is significantly higher in the Overberg and Central Karoo districts in 2019. Within the City of Cape Town, mortality varies across sub-districts likely driven mainly by socioeconomic inequities including access to health care. This geographic difference is consistent with nationally reported pneumonia mortality, which is made available annually [27, 28, 32].

### Strengths and limitations

This study was strengthened by the availability of comprehensive individual-level electronic health data with known causes of death. These study outcomes should enable policy makers and clinicians to better understand in-facility mortality due to infectious diseases over time and across geographies in the province using the de-duplicated, integrated dataset. In addition, the list of ICD-10 codes was comprehensive to ensure we were able to identify as many infectious disease hospitalisations and deaths as possible, allowing us to better understand the burden of disease in the community.

This study has several limitations. Firstly, only in-facility deaths were included based on the design and data availability. To understand the complete picture of child mortality, out-of-facility deaths would need to be included as well, however we did not have access to any data sources beyond DN for out-of-facility deaths. Secondly, we do not know if any in-facility deaths are missing as we were fully reliant on electronic capture of admissions and deaths. We believe that reporting of in-facility deaths may be more complete in recent years due to increases in networking of hospitals over time and the reduction in deaths is an underestimate, as a greater proportion of all deaths were reported electronically in more recent years. Thirdly, some errors in diagnostic coding are likely. However, this was mitigated as most primary diagnoses were coded by a registrar or consultant rather than a data clerk. Fourthly, the ICD-10 codes entered electronically were not verified against the in-facility written patient folders. We believe the impact of this was limited given: (i) diagnostic coding has been shown to be better in deceased children [8] and (ii) there was a high level of agreement of cause of death, particularly for diarrhoea and LRTI, when compared to the Child PIP and DN Surveillance. Fifthly, we did not have Child PIP and DN Surveillance for the entire time period of the study, which may result in infectious diseases being missed in RHIS, if they were identified as infectious in Child PIP or DN Surveillance. However, we believe this was mitigated by improved diagnostic coding in recent years and among children who die. Finally, the approach and results from comparing death data may not be generalisable beyond the Western Cape for several reasons: the Western Cape has different health information systems to other provinces and countries, many of which have not or only partially implemented a unique patient identifier. There may be differential practices for diagnostic coding and different case definitions across provinces for national reporting, and routinely collected data quality may be suboptimal.

## Conclusion

Our study demonstrated decreasing admission mortality rates across the province over time, with plateauing in recent years and good agreement of RHIS cause of death data with the more accurate child death audit and DN data, particularly for LRTI and diarrhoea. This validates the use of routine data systems such as the RHIS data in the Western Cape to understand mortality on a regular cadence, and highlights the importance of strengthening the collection and curation of this data. Nonetheless, routine health service data is strengthened by integrating with data from additional sources such as child death audit systems and DNs, emphasizing the value of both vital registration and routine child death audits. Data on cause of death from death certificates including out-of-facility deaths should be made available to health services so that they can accurately monitor child health and the outcomes of the services they deliver. Additionally, this approach of using RHIS or similar data systems and integrating different sources of death data could be extensible to other infectious and/or non-infectious diseases and/or populations in the Western Cape or other settings where data are either already available or can be strengthened and integrated.

## Supplementary Information

**Additional file 1: Table S1.** List of International Classification of Diseases 10th Revision (ICD-10) codes used for infectious disease coding.

## Data Availability

The data that support the findings of this study are available from the Western Cape Provincial Health Data Centre, but restrictions apply to the availability of these data, which were used under license for the current study, and so are not publicly available. Data are available, however, from the corresponding author upon reasonable request and with permission of the Western Cape Provincial Health Data Centre.
