# Supplementary material for: Comparison of paediatric infectious disease deaths in public sector health facilities using different data sources in the Western Cape, South Africa (2007–2021)
Source: BMC Infect Dis. 2023 Feb 22;23:104. doi: 10.1186/s12879-023-08012-6 (PMC9945739; doi:10.1186/s12879-023-08012-6)
Supplement: Supplementary file 1 — Additional file 1: Table S1. List of International Classification of Diseases 10th Revision (ICD-10) codes used for infectious disease coding. [file 12879_2023_8012_MOESM1_ESM.docx]

**Table S1.** List of International Classification of Diseases 10th Revision (ICD-10) codes used for infectious disease coding.

| **ICD-10 code** | **Description** |
| --- | --- |
| ***LRTI*** | |
| B01.2 | Varicella pneumonia (J17.1*) |
| B20.6 | HIV disease resulting in Pneumocystis jirovecii pneumonia |
| B25.0 | Cytomegaloviral pneumonitis (J17.1*) |
| B59 | Pneumocystosis (J17.3*) |
| J05 | Acute obstructive laryngitis [croup] and epiglottitis |
| J05.0 | Acute obstructive laryngitis [croup] |
| J05.1 | Acute epiglottitis |
| J05.X | Acute obstructive laryngitis [croup] and epiglottitis |
| J09 | Influenza due to identified zoonotic or pandemic influenza virus |
| J09.X | Influenza due to certain identified influenza virus |
| J10 | Influenza with other manifestations, other influenza virus identified |
| J10.0 | Influenza with pneumonia, other influenza virus identified |
| J10.1 | Influenza with other respiratory manifestations, other influenza virus identified |
| J10.8 | Influenza with other manifestations, other influenza virus identified |
| J10.X | Influenza due to other identified influenza virus |
| J11 | Influenza, virus not identified |
| J11.0 | Influenza with pneumonia, virus not identified |
| J11.1 | Influenza with other respiratory manifestations, virus not identified |
| J11.8 | Influenza with other manifestations, virus not identified |
| J11.X | Influenza, virus not identified |
| J12 | Viral pneumonia, not elsewhere classified |
| J12.0 | Adenoviral pneumonia |
| J12.1 | Respiratory syncytial virus pneumonia |
| J12.2 | Parainfluenza virus pneumonia |
| J12.3 | Human metapneumovirus pneumonia |
| J12.8 | Other viral pneumonia |
| J12.9 | Viral pneumonia, unspecified |
| J12.X | Viral pneumonia, not elsewhere classified |
| J13 | Pneumonia due to Streptococcus pneumoniae |
| J13.X | Pneumonia due to Streptococcus pneumoniae |
| J14 | Pneumonia due to Haemophilus influenzae |
| J14.X | Pneumonia due to Haemophilus influenzae |
| J15 | Bacterial pneumonia, not elsewhere classified |
| J15.0 | Pneumonia due to Klebsiella pneumoniae |
| J15.1 | Pneumonia due to Pseudomonas |
| J15.2 | Pneumonia due to Staphylococcus |
| J15.3 | Pneumonia due to Streptococcus, Group B |
| J15.4 | Pneumonia due to other streptococci |
| J15.5 | Pneumonia due to Escherichia coli |
| J15.6 | Pneumonia due to other aerobic Gram-negative bacteria |
| J15.7 | Pneumonia due to Mycoplasma pneumoniae |
| J15.8 | Other bacterial pneumonia |
| J15.9 | Bacterial pneumonia, unspecified |
| J15.X | Bacterial pneumonia, not elsewhere classified |
| J16 | Pneumonia due to other infectious organisms, not elsewhere classified |
| J16.0 | Chlamydial pneumonia |
| J16.8 | Pneumonia due to other specified infectious organisms |
| J16.X | Pneumonia due to other infectious organisms, not elsewhere classified |
| J17 | Pneumonia in diseases classified elsewhere |
| J17.0 | Pneumonia in bacterial diseases classified elsewhere |
| J17.1 | Pneumonia in viral diseases classified elsewhere |
| J17.2 | Pneumonia in mycoses |
| J17.3 | Pneumonia in parasitic diseases |
| J17.8 | Pneumonia in other diseases classified elsewhere |
| J17.X | Pneumonia in diseases classified elsewhere |
| J18 | Pneumonia, organism unspecified |
| J18.0 | Bronchopneumonia, unspecified |
| J18.1 | Lobar pneumonia, unspecified |
| J18.2 | Hypostatic pneumonia, unspecified |
| J18.8 | Other pneumonia, organism unspecified |
| J18.9 | Pneumonia, unspecified |
| J18.X | Pneumonia, organism unspecified |
| J20 | Acute bronchitis |
| J20.0 | Acute bronchitis due to Mycoplasma pneumoniae |
| J20.1 | Acute bronchitis due to Haemophilus influenzae |
| J20.2 | Acute bronchitis due to streptococcus |
| J20.3 | Acute bronchitis due to coxsackievirus |
| J20.4 | Acute bronchitis due to parainfluenza virus |
| J20.5 | Acute bronchitis due to respiratory syncytial virus |
| J20.6 | Acute bronchitis due to rhinovirus |
| J20.7 | Acute bronchitis due to echovirus |
| J20.8 | Acute bronchitis due to other specified organisms |
| J20.9 | Acute bronchitis, unspecified |
| J20.X | Acute bronchitis |
| J21 | Acute bronchiolitis |
| J21.0 | Acute bronchiolitis due to respiratory syncytial virus |
| J21.1 | Acute bronchiolitis due to human metapneumovirus |
| J21.8 | Acute bronchiolitis due to other specified organisms |
| J21.9 | Acute bronchiolitis, unspecified |
| J21.X | Acute bronchiolitis |
| J22 | Unspecified acute lower respiratory infection |
| J22.X | Unspecified acute lower respiratory infection |
| P23 | Congenital pneumonia |
| P23.0 | Congenital pneumonia due to viral agent |
| P23.1 | Congenital pneumonia due to Chlamydia |
| P23.2 | Congenital pneumonia due to staphylococcus |
| P23.3 | Congenital pneumonia due to Streptococcus, Group B |
| P23.4 | Congenital pneumonia due to Escherichia coli |
| P23.5 | Congenital pneumonia due to Pseudomonas |
| P23.6 | Congenital pneumonia due to other bacterial agents |
| P23.8 | Congenital pneumonia due to other organisms |
| P23.9 | Congenital pneumonia, unspecified |
| ***Diarrhoea*** | |
| A00.9 | Cholera, unspecified |
| A01 | Typhoid and paratyphoid fevers |
| A01.0 | Typhoid fever |
| A01.1 | Paratyphoid fever A |
| A01.2 | Paratyphoid fever B |
| A01.3 | Paratyphoid fever C |
| A01.4 | Paratyphoid fever, unspecified |
| A01.X | Typhoid and paratyphoid fevers |
| A02 | Other salmonella infections |
| A02.0 | Salmonella enteritis |
| A02.2 | Localized salmonella infections |
| A02.8 | Other specified salmonella infections |
| A02.9 | Salmonella infection, unspecified |
| A03 | Shigellosis |
| A03.0 | Shigellosis due to Shigella dysenteriae |
| A03.1 | Shigellosis due to Shigella flexneri |
| A03.2 | Shigellosis due to Shigella boydii |
| A03.3 | Shigellosis due to Shigella sonnei |
| A03.8 | Other Shigellosis |
| A03.9 | Shigellosis, unspecified |
| A03.X | Shigellosis |
| A04 | Other bacterial intestinal infections |
| A04.0 | Enteropathogenic Escherichia coli infection |
| A04.1 | Enterotoxigenic Escherichia coli infection |
| A04.2 | Enteroinvasive Escherichia coli infection |
| A04.3 | Enterohaemorrhagic Escherichia coli infection |
| A04.4 | Other intestinal Escherichia coli infections |
| A04.5 | Campylobacter enteritis |
| A04.6 | Enteritis due to Yersinia enterocolitica |
| A04.7 | Enterocolitis due to Clostridium difficile |
| A04.8 | Other specified bacterial intestinal infections |
| A04.9 | Bacterial intestinal infection, unspecified |
| A05 | Other bacterial foodborne intoxications, not elsewhere classified |
| A05.0 | Foodborne staphylococcal intoxication |
| A05.1 | Botulism |
| A05.2 | Foodborne Clostridium perfringens [Clostridium welchii] intoxication |
| A05.3 | Foodborne Vibrio parahaemolyticus intoxication |
| A05.4 | Foodborne Bacillus cereus intoxication |
| A05.8 | Other specified bacterial foodborne intoxications |
| A05.9 | Bacterial foodborne intoxication, unspecified |
| A06 | Amoebiasis |
| A06.0 | Acute amoebic dysentery |
| A06.1 | Chronic intestinal amoebiasis |
| A06.9 | Amoebiasis, unspecified |
| A07 | Other protozoal intestinal diseases |
| A07.0 | Balantidiasis |
| A07.1 | Giardiasis [lambliasis] |
| A07.2 | Cryptosporidiosis |
| A07.3 | Isosporiasis |
| A07.8 | Other specified protozoal intestinal diseases |
| A07.9 | Protozoal intestinal disease, unspecified |
| A08 | Viral and other specified intestinal infections |
| A08.0 | Rotaviral enteritis |
| A08.1 | Acute gastroenteropathy due to Norwalk agent |
| A08.2 | Adenoviral enteritis |
| A08.3 | Other viral enteritis |
| A08.4 | Viral intestinal infection, unspecified |
| A08.5 | Other specified intestinal infections |
| A08.X | Viral and other specified intestinal infections |
| A09 | Other gastroenteritis and colitis of infectious and unspecified origin |
| A09.0 | Other and unspecified gastroenteritis and colitis of infectious origin |
| A09.9 | Gastroenteritis and colitis of unspecified origin |
| A09.X | Other gastroenteritis and colitis of infectious and unspecified origin |
| K52.3 | Indeterminate colitis |
| R19.7 | Diarrhoea, unspecified |
| ***Meningitis*** | |
| ***Bacterial meningitis*** | |
| A20.3 | Plague meningitis |
| A32.1 | Listerial meningitis and meningoencephalitis |
| A39.0 | Meningococcal meningitis (G01*) |
| G00 | Bacterial meningitis, not elsewhere classified |
| G00.0 | Haemophilus meningitis |
| G00.1 | Pneumococcal meningitis |
| G00.2 | Streptococcal meningitis |
| G00.3 | Staphylococcal meningitis |
| G00.8 | Other bacterial meningitis |
| G00.9 | Bacterial meningitis, unspecified |
| G00.X | Bacterial meningitis, not elsewhere classified |
| G01 | Meningitis in bacterial diseases classified elsewhere |
| G01.X | Meningitis in bacterial diseases classified elsewhere |
| ***Viral meningitis*** | |
| A87 | Viral meningitis |
| A87.0 | Enteroviral meningitis (G02.0*) |
| A87.1 | Adenoviral meningitis (G02.0*) |
| A87.2 | Lymphocytic choriomeningitis |
| A87.8 | Other viral meningitis |
| A87.9 | Viral meningitis, unspecified |
| A87.X | Viral meningitis |
| B00.3 | Herpesviral meningitis (G02.0*) |
| B01.0 | Varicella meningitis (G02.0*) |
| B02.1 | Zoster meningitis (G02.0*) |
| B26.1 | Mumps meningitis (G02.0*) |
| G02.0 | Meningitis in viral diseases classified elsewhere |
| G03.0 | Nonpyogenic meningitis |
| G03.2 | Benign recurrent meningitis [Mollaret] |
| ***Fungal meningitis*** | |
| B37.5 | Candidal meningitis (G02.1*) |
| B38.4 | Coccidioidomycosis meningitis (G02.1*) |
| G02.1 | Meningitis in mycoses |
| ***Other meningitis*** | |
| G02 | Meningitis in other infectious and parasitic diseases classified elsewhere |
| G02.8 | Meningitis in other specified infectious and parasitic diseases classified elsewhere |
| G03 | Meningitis due to other and unspecified causes |
| G03.1 | Chronic meningitis |
| G03.8 | Meningitis due to other specified causes |
| G03.9 | Meningitis, unspecified |
| G03.X | Meningitis due to other and unspecified causes |
| ***Tuberculous meningitis*** | |
| A17 | Tuberculosis of nervous system |
| A17.1 | Meningeal tuberculoma (G07*) |
| A17.8 | Other tuberculosis of nervous system |
| A17.9 | Tuberculosis of nervous system, unspecified (G99.8*) |
| A17.X | Tuberculosis of nervous system |
| ***Other infectious disease*** | |
| B23.8 | HIV disease resulting in other specified conditions |
| D64.9 | Anaemia, unspecified |
| E86 | Volume depletion |
| G41.9 | Status epilepticus, unspecified |
| P28.1 | Other and unspecified atelectasis of newborn |
| R11 | Nausea and vomiting |
